# Prone Position in Management of COVID-19 Patients; a Commentary

**Published:** 2020-04-11

**Authors:** Parisa Ghelichkhani, Maryam Esmaeili

**Affiliations:** 1 Department of Intensive Care Nursing, School of Nursing and Midwifery, Tehran University of Medical Sciences, Tehran, Iran.; 2 Nursing and Midwifery Care Research Center, School of Nursing and Midwifery, Tehran University of Medical Sciences, Tehran, Iran.

**Keywords:** COVID-19, severe acute respiratory syndrome coronavirus 2, sars-cov-2, prone position, respiratory distress syndrome, adult, pandemics

## Abstract

SARS-CoV-2 virus causes a pneumonia that was identified through fever, dyspnea, and acute respiratory symptoms and named COVID-19. This disease exacerbates in a number of patients and causes pulmonary edema, multi-organ failure, and acute respiratory distress syndrome (ARDS). Prevalence of ARDS among COVID-19 patients has been reported to be up to 17%. Among the introduced treatment methods for management of ARDS patients, prone position can be used as an adjuvant therapy for improving ventilation in these patients. Here we reviewed the literature regarding the role of prone position in management of COVID-19 patients.

**Dear editor:**

In late 2019, a new virus was introduced to the world, which caused COVID-19. The virus rapidly spread all over the world and led to a high rate of mortality and became a great challenge for the healthcare staff. SARS-CoV-2 virus causes a pneumonia that was identified through fever, dyspnea, and acute respiratory symptoms and named COVID-19. (1). This disease exacerbates in a number of patients and causes pulmonary edema, multi-organ failure, and acute respiratory distress syndrome (ARDS). Prevalence of ARDS among COVID-19 patients has been reported to be up to 17% (2).

ARDS was first introduced in 1968 with clinical presentations such as acute hypoxemia, non-cardiac pulmonary edema, decrease in pulmonary compliance, and increase in work of breathing. It was especially seen in patients who had an underlying sepsis, pneumonia, and aspiration or severe trauma and all of these patients were in need of positive pressure ventilation (3). 10% of patients who are admitted to the intensive care unit (ICU) develop ARDS (3) and despite all the treatment advances made, the rate of mortality is still high among these patients and has been reported to be between 30% and 40% (3).

Among the introduced treatment methods for management of ARDS patients, prone position can be used as an adjuvant therapy for improving ventilation in these patients. It should be prescribed along with low tidal volume (6 cc per kg body weight) and infusion of neuromuscular blockers (cisatracurium for 48 hours). These 3 treatment strategies together, lead to improvement in oxygenation and survival of ARDS patients (4). 

The main mechanisms of prone position in improvement of ARDS patients’ condition are affecting recruitment in dorsal lung regions, increasing end-expiratory lung volume, increasing chest wall elastane, decreasing alveolar shunt, and improving tidal volume (5). Patients remaining in lengthy prone position sessions leads to decrease in mortality of patients (6). However, correct selection of patients and applying the proper treatment protocol for prone positioning are key to its effectiveness. For instance, in a meta-analysis, Munshi et al. expressed that prone position can lead to a drop in the rate of mortality among patients with severe ARDS when applied to patients for least 12 hours a day (7). Additionally, in another meta-analysis it was revealed that prone position can only reduce mortality due to ARDS when patients are ventilated with low tidal volume, the treatment is started within the initial 48 hours of initiation of the disease, and patients have severe hypoxia. In other words, prone position can reduce mortality only when prescribed for patients with severe impaired oxygenation, in the initial hours, and for long durations (8).

Recently, in a multi-centered observational study, Guérin et al. showed that only 13.7% of patients with ARDS have been placed in prone position. Even in patients with severe ARDS, the rate of using this technique was 32.9%. In the mentioned study, 2 main reasons were given for the physicians’ reluctance to use this treatment method: 1- Based on the judgment of the physicians in most cases, the hypoxia in severe ARDS patients is not severe enough to justify using prone position. 2- Most ARDS patients have hemodynamic instability, which prevents the physicians from deciding to use prone position (9).

In addition to the effectiveness of this treatment method, caretaking aspects and the side effects of this position on ARDS patients should also be considered. Patients that undergo ventilation with ventilator in prone position face risks such as accidental removal of the tracheal tube, limited access to the venous route, bending or pulling of the catheters and chest tube, pressure wound, bruising around the mouth due to presence of the tracheal tube, edema around the eyes and facial edema, Gastroesophageal reflux, hyper-salivation and skin injuries (10). In prone position, the patient should face the ventilator and in patients with tracheostomy, a roll of fabric or pillow should be placed under the shoulders to prevent airway obstruction, these patients should receive muscle and nerve relaxant medications and high-dose sedation as infusion, eye pads should be used for closing the patients’ eyes to prevent corneal ulcers. Considering the condition of these patients and presence of pressure on their stomach, the probability of reflux after gavage is very high, so they must be closely monitored regarding aspiration of gastric contents (10).

The position of patients placed in prone position should be changed every 2 hours and sides should be switched. At least 3 to 5 individuals should participate to correctly put intubated patients in prone position, which is a serious limitation for keeping the patient in this position for a long time. To solve this problem a tool called Vollman has been introduced for facilitation of moving patients placed in this position to prevent pressure wounds and deformity of joints (11).

Overall, it seems that studies on the effectiveness of prone position in ARDS patients clearly point out that correct patient selection, timely initiation and duration of patient’s placement in this position can all affect the effectiveness of this treatment method. Available meta-analyses show that prone position can decrease mortality in ARDS patients when performed in the initial hours of disease manifestation, in patients with severe impaired oxygenation and for a long time (8). The minimum suggested duration of prone position is 12 hours a day.
